# On marker-based parentage verification via non-linear optimization

**DOI:** 10.1186/s12711-017-0324-3

**Published:** 2017-06-15

**Authors:** Vinzent Boerner

**Affiliations:** Animal Genetics and Breeding Unit, University of New England, Armidale, 2351 Australia

## Abstract

**Background:**

Parentage verification by molecular markers is mainly based on short tandem repeat markers. Single nucleotide polymorphisms (SNPs) as bi-allelic markers have become the markers of choice for genotyping projects. Thus, the subsequent step is to use SNP genotypes for parentage verification as well. Recent developments of algorithms such as evaluating opposing homozygous SNP genotypes have drawbacks, for example the inability of rejecting all animals of a sample of potential parents. This paper describes an algorithm for parentage verification by constrained regression which overcomes the latter limitation and proves to be very fast and accurate even when the number of SNPs is as low as 50. The algorithm was tested on a sample of 14,816 animals with 50, 100 and 500 SNP genotypes randomly selected from 40k genotypes. The samples of putative parents of these animals contained either five random animals, or four random animals and the true sire. Parentage assignment was performed by ranking of regression coefficients, or by setting a minimum threshold for regression coefficients. The assignment quality was evaluated by the power of assignment (P$_{\text {a}}$) and the power of exclusion (P$_{\text {e}}$).

**Results:**

If the sample of putative parents contained the true sire and parentage was assigned by coefficient ranking, P$_{\text {a}}$ and P$_{\text {e}}$ were both higher than 0.99 for the 500 and 100 SNP genotypes, and higher than 0.98 for the 50 SNP genotypes. When parentage was assigned by a coefficient threshold, P$_{\text {e}}$ was higher than 0.99 regardless of the number of SNPs, but P$_{\text {a}}$ decreased from 0.99 (500 SNPs) to 0.97 (100 SNPs) and 0.92 (50 SNPs). If the sample of putative parents did not contain the true sire and parentage was rejected using a coefficient threshold, the algorithm achieved a P$_{\text {e}}$ of 1 (500 SNPs), 0.99 (100 SNPs) and 0.97 (50 SNPs).

**Conclusion:**

The algorithm described here is easy to implement, fast and accurate, and is able to assign parentage using genomic marker data with a size as low as 50 SNPs.

## Background

The advent of DNA markers has facilitated verification of candidate parents thus enabling more accurate pedigrees for genetic evaluation, resolution of conflicts in the trading of breeding animals, and basic parent identification in extensive production systems. For the last two decades, this verification was based on short tandem repeat markers (STR), which are commonly called micro-satellites, are highly polymorphic and thus allow discrimination between individuals even when the total number of markers used is small. Due to their highly polymorphic character, parentage assignment on the basis of STR can be done by simple exclusion or by categorical allocation. For an exhaustive review of parentage assignment algorithms see [1].

However, in the last decade bi-allelic single nucleotide polymorphisms (SNPs) have quickly become the marker of choice for genotyping projects. Their sheer abundance has made them much more suitable for genome-wide association studies than other polymorphic markers. Furthermore, imputation techniques provide a compatibility layer between different SNP genotypes relieving researchers of the necessity to regenotype ancient animals as SNP panels change. Gradually, SNP genotypes have become the backbone of genomic selection which is now replacing pedigree selection in many livestock industries [2].

Animal breeding is done within an economic environment, and it was only a matter of time before questions arose about the need to genotype animals twice, with STR for parentage verification and with SNPs for genomic selection. Since many ancient animals have been genotyped with STR only, a first step to merge both approaches was to impute STR from SNP genotypes [3]. While this provides the necessary compatibility link between STR-based parentage verification and SNP genotypes during a transition period, parentage verification should omit imputation and rely on SNPs once SNP genotypes for both parents and offspring are available. However, compared to STR the bi-allelic nature of SNPs requires many more markers for a successful parent identification and based on results from simulations and real data it was suggested that between 50 to 500 markers were necessary [4–7]. In addition, it has been necessary to develop new algorithms that could exploit information from SNP genotypes for that purpose. One method counts the number of opposing homozygous marker loci (OHL), and the result is used as a possible measure of parentage [8, 9]. With this method, parents are identified as having the smallest number of loci with a homozygosity status opposite to that of the offspring because opposing homozygosity between parents and offspring is theoretically impossible, but can result from genotyping errors. Although improvements in the computational efficiency have made application of the method practical [10], its main shortcoming remains, which is that the sample of putative parents must contain one or two true parents, and knowledge about how many true parents are in the sample must exist [11]. Likelihood-based methods (LH) [12, 13], which were originally developed to use STR, can allow for the absence of the true parents but are slow. Modified likelihood methods have been developed for application to SNP genotypes but have difficulty assigning parents when the number of SNPs is small (100 or less) [11].

This article describes a non-linear optimisation approach for parentage assignment, called “constrained genomic regression” (CGR), which overcomes the limitation of OHL counting. The algorithm was tested on a data set of 19,051 Australian Angus beef cattle SNP genotypes which contained 14,816 sire-offspring pairs. CGR results were compared to results from parentage assignment via OHL counting [8, 9] and to LH-based parentage assignment implemented in the publicly available software “Cervus” [12].

## Methods

### Model

The problem to solve can be written as a simple linear regression equation:

1 $$\begin{aligned} \mathbf{y}= {} {\mathbf{Xb}} + \mathbf{e} \end{aligned},$$

where **y** is the vector of marker genotypes of the animal with uncertain parentage (explained animal), **X** is a column matrix of marker genotypes of the possible parents (explanatory animals), and **e** is the vector of residuals which cannot be explained. Columns in **X** can be genotypes of single animals (e.g. sire or dam), or functions of genotypes of single animals or several animals (e.g. population allele frequencies or expected gene content). Values in vector **b** are regression coefficients regressing **y** on the columns in **X**.

Assuming that the variance of **y** is equal to **I** $\sigma ^2$, minimising **e’e** would yield an ordinary least square solution. Since the parameter space of values in **b** is unconstrained, regression coefficients may become negative. To avoid this, the parameter space of **b** can be constrained to the interval between zero and $\infty$, and the sum over **b** can be constrained to be equal to 1. Thus, Eq. 1 becomes:

2 $$\begin{aligned}\mathop {{{\mathrm{arg\,min}}}}\limits _b f(\mathbf{b}) = \mathbf{y}'\mathbf{y} - 2\mathbf{y}'\mathbf{X}\mathbf{b} + \mathbf{b}'\mathbf{X}'\mathbf{X}\mathbf{b} \end{aligned}$$

3 $$\begin{aligned}&\text {subject to}\nonumber \\ &\quad b_i \ge 0\ \{i=1,\ldots ,N\} \end{aligned}$$

4 $$\begin{aligned}\sum _i^Nb_i = 1 \end{aligned}.$$

Minimising Eq. 2 with respect to **b** constrained by Eqs. 3 and 4 yields a vector **b** with values that explain the genotype in **y** as a linear function of genotypes in **X**. Coefficients in **b** are interpreted as the proportion of values in vector **Xb** explained by each column in **X**. Note that only the combination of constraints 3 and 4 guarantees that coefficients in **b** have this meaning.

If **X** contained genotypes of putative sires and dams only, values in **b** are not guaranteed to give unambiguous results for parentage assignment. Thus, it is highly advisable to always add to **X** the vector of population allele frequencies, or depending on the allele coding, the vector of expected gene content.

### Data

Genotypes of 19,051 animals of the Australian Angus beef cattle breed were used as a test data set which included 14,816 sire-offspring pairs (thus multiple progeny per sire). The genotypes were obtained from the Australian Beef Cooperative Research Center (www.beefcrc.com, Beef CRC) database and from cooperating breeders using the Illumina 50K Bead Chip, and were coded as “0” and “2” for the homozygous genotypes and “1” for the heterozygous genotype. After quality control, 40,627 SNPs were used in the analysis.

#### Configuration of genotypes

Subsets of 500, 100 and 50 SNPs were randomly selected from the full 40k genotypes. To ensure sufficient contrast between individual genotypes, the sample space for the 100 and 50 SNP set was restricted to those SNPs with a minor allele frequency higher than 0.3.

#### Animal assignment to the equation

From the full set of 19,051 animals, 14,816 animals were selected which had a genotyped sire in the data set. These animals will be called “explained animals” in the remainder of the article. For each of these animals, a unique Eq. 1 was formed where the genotype was used to construct the **y** vector. Matrix **X** always contained six columns, but they were filled in two different ways: (1) two columns for the genotype of the known sire and the vector of expected gene contents, and the remaining columns for the genotypes of a set of four randomly selected animals ($\mathbf{X}_{\mathbf{sire}}$), and (2) one column for the vector of expected gene contents and the remaining columns for the genotypes of a set of five randomly selected animals ($\mathbf{X}_{\mathbf{ran}}$). Columns in **X** might be interpreted as a pool of putative parents. This pool will be called “explanatory animals” in the remainder of the article.

Note that the randomly selected animals excluded parents, offspring, full sibs and half sibs of the animal in **y**, and were re-sampled for every **y**. Furthermore, the allele coding required use of the expected gene content rather than the allele frequency.

### Parentage assignment

Two different methods were used to assign parentage to individuals in **X**. The first ($\text {CGR}_\mathrm{R}$) ranked the coefficients in **b** after excluding the coefficient for the expected gene content. The sire was the individual with the greatest coefficient. Note that this method is similar to ranking of OHL counting results and does not allow for the rejection of all putative parents. It also requires knowledge about the number of true parents in the data set. It was therefore used only for the test runs using $\mathbf{X}_{\mathbf{sire}}$.

The second method ($\text {CGR}_\mathrm{T}$) requires setting a minimum threshold for the coefficients in **b**. Every animal which has a coefficient below this threshold is ruled out as a potential parent. If the threshold is set appropriately (higher than $\frac{1}{3}$), constrained Eq. 4 will ensure that the number of explanatory animals with coefficients higher than the threshold is lower or equal to 2, thus avoiding parentage over-assignment.

### Assignment statistics

Coefficients in **b** were summarised within each combination of SNP genotype set and **X** matrix in terms of means, standard deviations, minimum and maximum for single columns in **X** (sire, expected gene content) and for the sub-matrix in **X** containing the randomly chosen animals.

In addition, the power of assignment (P$_{\text {a}}$) was calculated as:

5 $$\begin{aligned} \frac{\text {number of correct parent assignments}}{\text {maximum number of true parents}} \end{aligned},$$

where the denominator was 14,816, which is the total number of true parents in the data set when $\mathbf{X}_{\mathbf{sire}}$ was used. The power of exclusion (P$_{\text {e}}$) was calculated as:

6 $$\begin{aligned} 1-\frac{\text {number of wrong parent assignments}}{\text {maximum number of parents}} \end{aligned},$$

where the denominator was 29,632, which is twice the number of progeny or the total number of possible parents.

### Parentage verification based on counting opposing homozygous loci and likelihood evaluation

CGR results were compared to OHL counting [8, 9] and a LH-based methodology [12]. Since OHL counting requires that the sample of putative parents contains at least one true parent, this method was applied to the $\mathbf{X}_{\mathbf{sire}}$ set only.

### Software

CGR was implemented in a FORTRAN program which called the NLopt library [14]. The optimisation solver used the augmented Lagrangian algorithm as a global solver and the method of moving asymptotes as a local solver. Note that the NLopt library is also available as an R package. Thus, the interested reader may implement the above methodology in a simple R script.

For LH-based parentage verification, the publicly available software “Cervus” was used [12]. Cervus requires three steps: (1) allele frequency analysis, (2) parentage simulation, and (3) parentage assignment. Parameters for step 2 were set as follows: type = “parent pair with unknown sex”, number of offspring = 10,000, number of candidate parents per offspring = 5, proportion of parents sampled = 1, proportion of loci typed = 1, proportion of loci mistyped = 0.01, confidence is calculated using = delta, confidence levels = 80%(relaxed) and 95%(strict). Parameters for step 3 were set as follows: type = “parent pair with unknown sex”. Note that the latter analysis type was chosen because it does not require prior knowledge about the data set.

Cervus results were evaluated for each **y** only for the parent pair with the highest lod score. For the $\mathbf{X}_{\mathbf{sire}}$ data set, two cases were distinguished: Case (A) the lod score was not significant. If the true sire was in the parent pair, the numerator of Eq. 5 was increased by one. Case (B) the lod score was significant. If the true sire was in the parent pair, the numerators of Eqs. 5 and 6 were increased by one. If the true sire was not in the parent pair, the numerator of Eq. 6 was increased by two. For $\mathbf{X}_{\mathbf{ran}}$ data set, the numerator of Eq. 6 was increased by 2 for every significant parent pair only.

All computations were carried out on a computer with an Intel(R) Core(TM) i7-3770 processor and 32GB of memory.

### Results

#### Parentage assignment

Tables 1 and 2 summarise the results for parentage assignment using different SNP genotypes and different pools of explanatory animals. For the $\text {CGR}_\mathrm{T}$ algorithm, the threshold to assign parentage was set to $\frac{1}{3}$. Descriptive statistics for the regression coefficients are in Table 1.

**Table 1:** Mean (${\overline{\text {x}}}$), standard deviation (s), minimum (min) and maximum (max) of the regression coefficients

Coefficient	$\hbox {X}_{\mathrm{sire}}$				$\hbox {X}_{\mathrm{ran}}$			
	$\overline{\text {x}}$	s	Min	Max	mean	s	Min	Max
Sire	0.492	0.065	0.000	0.764	–	–	–	–
Ran	0.018	0.028	0.000	0.337	0.022	0.035	0.000	0.413
Mean	0.435	0.085	0.034	1.000	0.891	0.077	0.389	1.000
Sire	0.492	0.091	0.000	0.831	–	–	–	–
Ran	0.036	0.054	0.000	0.478	0.042	0.063	0.000	0.535
Mean	0.363	0.139	0.000	1.000	0.789	0.141	0.000	1.000
Sire	0.492	0.119	0.000	0.904	–	–	–	–
Ran	0.050	0.074	0.000	0.548	0.059	0.087	0.000	0.773
Mean	0.308	0.177	0.000	1.000	0.704	0.197	0.000	1.000

Number of SNPs used as genotypes: upper part = 500 SNPs, middle part=100 SNPs and lower part = 50 SNPs. $\hbox {X}_{\mathrm{sire}}$: the sample of the putative parents contained the true sire, four randomly selected animals and the vector of expected gene contents. $\hbox {X}_{\mathrm{ran}}$: the sample of the putative parents contained five randomly selected animals and the vector of expected gene contents. sire: statistics for the coefficients regressing the focused animal on the genotype of the true sire. mean: statistics for the coefficients regressing the focused animal on the vector of expected gene contents. ran: statistics for the coefficients regressing the focused animal on randomly selected animals. The number of random animals was 4 when the sample of putative parents contained the true sire, and 5 otherwise

When 500 randomly selected SNPs were used as genotypes and the sample of putative parents contained the true sire, P$_{\text {a}}$ of all algorithms was higher than 0.99 with marginal differences between the methods. Thus, $\text {CGR}_\mathrm{R}$, $\text {CGR}_\mathrm{T}$, OHL counting and LH detected the true sire equally well. When the sample of putative parents did not contain the true sire, $\text {CGR}_\mathrm{T}$ and LH successfully rejected the randomly selected animals as parents with $\text {CGR}_\mathrm{T}$ resulting in four and LH in zero incorrect assignments out of 29,632 possible assignments (see Table 2, upper part).

Decreasing marker density to 100 SNPs randomly selected from those which had a minor allele frequency higher than 0.3 made the correct assignment more difficult (see Table 2, middle part). When the sample of putative parents contained the true sire, the best results were achieved by LH which detected 14,735 true sires correctly, followed by $\text {CGR}_\mathrm{R}$ with 14,711 and OHL counting with 14,699 correctly assigned sires. It should be noted that OHL counting, as well as $\text {CGR}_\mathrm{R}$, will automatically assign a wrong parent if the true parent is missed. The same data set yielded the opposite ranking of algorithms when parameter P$_{\text {e}}$ was evaluated. $\text {CGR}_\mathrm{T}$ achieved the best result with only 15 incorrectly assigned parents, followed by $\text {CGR}_\mathrm{R}$ (105), OHL counting (117) and LH (213). When the set of putative parents did not contain the true sire, LH performed best rejecting all random animals as parents, whereas $\text {CGR}_\mathrm{T}$ assigned incorrect parentage in 96 cases, resulting in a P$_{\text {e}}$ of 0.997.

A further decrease in marker density to 50 SNPs randomly selected from those which had a minor allele frequency higher than 0.3 yielded the same algorithm ranking as the 100 SNP data set, but with slightly deteriorated P$_{\text {a}}$ and P$_{\text {e}}$ values for all algorithms (see Table 2, lower part). When the set of putative parents contained the true sire the decrease in P$_{\text {a}}$ was strongest for $\text {CGR}_\mathrm{T}$ (0.918), followed by OHL counting (0.978), $\text {CGR}_\mathrm{R}$ (0.983) and LH (0.988). P$_{\text {e}}$ ranked the algorithms inversely with LH (0.954) showing the strongest deterioration, followed by OHL counting (0.989), $\text {CGR}_\mathrm{T}$ (0.991) and $\text {CGR}_\mathrm{R}$ (0.992). LH assigned five times more often false parentage than $\text {CGR}_\mathrm{R}$. However, when the set of putative parents contained only random animals, LH assigned false parentage in 40 cases, whereas $\text {CGR}_\mathrm{T}$ assigned 960 parents falsely.

**Table 2:** Power of assignment (Pa) and power of exclusion (Pe)

Algorithm	$\hbox {X}_{\mathrm{sire}}$		$\hbox {X}_{\mathrm{ran}}$	
	Pa	Pe	Pa	Pe
$\text {CGR}_\mathrm{T}$	0.990 (14,664)	1.000 (1)	–	1 (4)
$\text {CGR}_\mathrm{R}$	0.994 (14,730)	0.997 (86)	–	–
OHL counting	0.993 (14,717)	0.997 (99)	–	–
LH	0.995 (14,744)	1.000 (5)	–	1 (0)
$\text {CGR}_\mathrm{T}$	0.969 (14,361)	0.999 (15)	–	0.997 (96)
$\text {CGR}_\mathrm{R}$	0.993 (14,711)	0.996 (105)	–	–
OHL counting	0.992 (14,699)	0.996 (117)	–	–
LH	0.995 (14,735)	0.993 (213)	–	1.000 (0)
$\text {CGR}_\mathrm{T}$	0.918 (13,607)	0.991 (252)	–	0.968 (960)
$\text {CGR}_\mathrm{R}$	0.983 (14,570)	0.992 (246)	–	–
OHL counting	0.978 (14,489)	0.989 (327)	–	–
LH	0.988 (14,639)	0.954 (1373)	–	0.999 (40)

The numerator of the related equations is given in brackets. Number of SNPs used as genotypes: upper part = 500 SNPs, middle part = 100 SNPs and lower part = 50 SNPs. $\hbox {X}_{\mathrm{sire}}$: the sample of the putative parents contained the true sire, four randomly selected animals and the vector of expected gene contents. $\hbox {X}_{\mathrm{ran}}$: the sample of the putative parents contained five randomly selected animals and the vector of expected gene contents. Pa: probability of assigning the right parent if the sample of putative parents contained the true sire. Pe: probability of rejecting the wrong parent in favour of the right parent or the vector of expected gene contents. SNPs were randomly selected from 40k genotypes with the sample space for the 100 and 50 sets restricted to those SNPs with a minor allele frequency ${>}0.3$

#### Computational demand

Besides reading the data, CGR solving time for Eq. 2 for all 14,816 animals was 18 real time seconds irrespective of the number of SNPs used, which is 0.001 real time seconds per animal. Note that using all 40k SNPs increased the solving time only marginally to 21 real time seconds. The LH method implemented in Cervus needed 180, 51 and 35 real time seconds for the 500, 100 and 50 SNP data sets, respectively. OHL counting was not evaluated in terms of speed but can be assumed to provide results most quickly.

## Discussion

Results show that parentage can be successfully assigned with as few as 500 SNPs using $\text {CGR}_\mathrm{R}$, $\text {CGR}_\mathrm{T}$, OHL counting or a likelihood-based method, and for which all four approaches perform equally well. However, OHL counting, and therefore $\text {CGR}_\mathrm{R}$ as well, are less suitable for practical applications because they are incapable of rejecting all putative parents [11].

The above result also holds for the 100 SNP data set with which $\text {CGR}_\mathrm{R}$, OHL counting and LH maintained P$_{\text {a}}$ and P$_{\text {e}}$ values higher than 0.99, and $\text {CGR}_\mathrm{T}$ achieved 0.97 and 0.99, respectively. With a further decrease in the number of SNPs, all four algorithms had some difficulties, but P$_{\text {a}}$ and P$_{\text {e}}$ remained generally higher than 0.98. However, P$_{\text {a}}$ for $\text {CGR}_\mathrm{T}$ and P$_{\text {e}}$ for LH decreased to 0.92 and 0.95, respectively.

The performance of OHL counting should be regarded cautiously because the range of OHL counts between the true sire and its offspring was between 0 and 10 when using the 50 SNP genotypes, with 1013 cases for which the contrast between the true sire and the random animal ranked next differed by a single count. This contrast might be by chance only and be missed in a future genotype sample, rendering the assignment statistics of OHL counting the worst of all four algorithms. Moreover, constructing an OHL matrix between all 19,051 individuals with 50 SNP genotypes as given in [10] and evaluating the respective matrix rows of the 14,816 test animals revealed that 14,782 animals had at least one zero entry in addition to the diagonal and sire entry. The most frequent zero count was 11 which occurred for 294 test animals. Thus, there is a certain probability that a sample of putative parents may contain an animal for which the genotype will yield a lower OHL count than the genotype of the true parent. Besides these rather sobering insights into the superficially very good performance of OHL counting, it is also undesirable to use a method based on genotyping errors, which should be minimised and ultimately eradicated. The basic implementation of the method will always assign one or two parents depending on prior information about the data and cannot reject all candidate parents. OHL counting could be made more versatile by generating an empirical distribution of OHL counts by simulation which is then used as a test statistic. However, such a distribution will be genotype sample dependent and its generation requires knowledge about the genotyping error probability, which in turn can only be estimated if trios of offspring and both true parents are known.

When the true parent was in the sample, the LH-based method implemented in the software “Cervus” performed as well as OHL counting and $\text {CGR}_\mathrm{R}$ across all three SNP data sets. By contrast, it gave the worst results in terms of rejecting non-parents for the 100 SNP and 50 SNP data sets. This poor performance may result from the LH software being set to the analysis type “parent pair with unknown sex” with the combined likelihood of one true and one false parent being still high enough to assign parentage to both. Changing the analysis type may increase the performance, but would require prior knowledge about the data set which is not guaranteed to be available in practical applications. In addition, the LH method has two major drawbacks compared to the three other algorithms: scaleability and, as a direct result, processing time. For the 500 SNP data set, the latter was 10 times slower than that of the CGR algorithms. Although this difference might be negligible given the absolute processing time, it may become prohibitive if parentage must be verified for hundreds of thousands of animals. In addition, situations may arise where results from small SNP genotypes may be disputed and need to be verified using more markers, e.g. up to full 40k genotypes. In such cases, processing time may render the LH approach impractical.

By contrast, processing time for CGR increased only marginally when the full 40k genotypes were used. Moreover, unlike LH, the two implementations of CGR tested do not require any pre-analysis simulation. While $\text {CGR}_\mathrm{R}$ suffers the same drawback as OHL counting, $\text {CGR}_\mathrm{T}$ is able to reject all putative parents. The ability of $\text {CGR}_\mathrm{T}$ to contrast between true and false parents may be further enhanced by testing coefficients in **b** against their empirical distribution. This may help to enhance performance when using only very few SNPs (e.g. the 50 SNP data set), but would require pre-analysis simulations. However, since practical parentage verification aims at using at least 500 SNPs [7], the results suggest that the imposed threshold on values in **b** is sufficient to identify parents correctly. CGR may also account for genotyping errors by substituting the observed genotype at a given locus by its expected value after accounting for the genotyping error probability.

It is perhaps unexpected that the rather general approach of CGR performs as well as the elaborate LH methodology. For the sake of simplicity, one may consider a situation where all allele frequencies are equal to 0.5. Subtracting the vector of expected gene content (allele frequencies) from the columns in **X** and from **y** will set all heterozygous loci to zero as well as the column in **X** containing the expected gene content. This reveals that the optimum solution for **b** depends only on the homozygosity status of the putative parents. In addition, when allele frequencies deviate from 0.5, parent-offspring homozygosity for rare alleles will have a larger contribution than for very common alleles. Interestingly, the LH method exploits little more information. The biggest contribution to the likelihood comes from the sire-dam-offspring genotype combinations 2-2-2, 0-0-0, 2-0-1 and 0-2-1, which all contribute to the likelihood with a coefficient of 1. Excluding genotyping errors, all other possible combinations yield a coefficient of 0.5, and therefore provide no contrast between putative parents. The only two exceptions are the combinations 1-1-0 and 1-1-2 with a coefficient of 0.25, which reduces the likelihood and increases the contrast between putative parents. The LH method exploits the information content of rare alleles by scaling the sire-dam-offspring probability by the offspring genotype probability calculated from the population allele frequencies. As already pointed out above, CGR makes use of this information as well.

The test data sets used in this analysis were limited to five putative parents per progeny. In practical applications, it might be necessary to search the whole genotyped population for possible parents which could be achieved by expanding the number of columns of matrix **X** to all genotyped animals. However, if the number of SNPs is small, the system in Eq. 1 will be over-parameterised. In addition, processing time is likely to be incompatible. In a single test run using all 40k SNPs and **X** having more than 19,000 columns, the correct parent was identified by $\text {CGR}_\mathrm{T}$ and $\text {CGR}_\mathrm{R}$ but the processing time was 23 min. The formulas in Eqs. 1 and 2 imply that CGR uses sub-matrices and vectors from an uncentered and unscaled genomic relationship matrix (GRM). Thus, as an alternative to expanding **X** to all genotyped animals, one may decrease the number of putative parents in a pre-analysis step to those which have the largest off-diagonal values in the GRM row related to the animal in **y**. These animals are then used to construct the columns in **X**.

## Conclusions

CGR is a fast, efficient, accurate and easy to implement algorithm to assign parentage on the basis of SNP genotypes in samples which contain at least one true parent, or to reject parentage if the samples do not contain a true parent at all. CGR scales automatically to any size of genotypes and has proven to give accurate results with genotypes based on 50 SNPs only.
